# Platelet-rich plasma in reproductive medicine: from ovarian follicles to the ovarian niche and emerging stem cell mobilization–based regenerative strategies

**DOI:** 10.3389/frph.2026.1921016

**Published:** 2026-08-14

**Authors:** Manuel Muñoz-Cantero, Amparo Santamaría Ortiz

**Affiliations:** IVI RMA Global, IVI Alicante, Alicante, Spain

**Keywords:** diminished ovarian reserve, G-CSF, ovarian aging, ovarian niche, ovarian niche restoration, ovarian regeneration, ovarian rejuvenation, Platelet-rich plasma (PRP)

## Abstract

Ovarian aging is a complex biological process traditionally attributed to the progressive depletion of the follicular reserve. However, accumulating evidence suggests that age-related reproductive decline is not solely determined by follicular loss but also by profound alterations in the ovarian microenvironment, including stromal fibrosis, vascular dysfunction, chronic low-grade inflammation, oxidative stress, and impaired intercellular communication. This evolving understanding has promoted a shift from a follicle-centered view of ovarian aging toward a broader ovarian niche–centered paradigm. Within this context, platelet-rich plasma (PRP) has emerged as one of the most extensively investigated regenerative approaches in reproductive medicine. PRP provides a concentrated source of growth factors, cytokines, and bioactive mediators capable of modulating angiogenesis, tissue remodeling, inflammation, and cellular signaling. Nevertheless, interpretation of the available clinical evidence remains challenging due to substantial heterogeneity in PRP preparation protocols, biological composition, patient selection, and outcome measures. This narrative review examines the biological basis of PRP, its potential effects on the ovarian niche, and the current clinical evidence supporting its use in women with diminished ovarian reserve, poor ovarian response, premature ovarian insufficiency, and advanced reproductive age. Particular attention is given to the emerging concept that restoration of ovarian niche functionality, rather than isolated activation of residual follicles, may represent the primary biological objective of contemporary ovarian rejuvenation strategies. The review also explores the rationale for combining local regenerative interventions with systemic regenerative approaches, including hematopoietic progenitor cell mobilization induced by granulocyte colony-stimulating factor (G-CSF). We discuss the hypothesis that mobilization-induced changes in circulating progenitor cells, chemotactic signaling pathways, and the systemic regenerative milieu may enhance the biological activity of autologous regenerative products and contribute to ovarian niche restoration. Current evidence does not yet support definitive conclusions regarding the efficacy of ovarian rejuvenation therapies. However, the integration of local and systemic regenerative signals represents a promising avenue for future investigation. Further progress will require standardized biological products, robust biomarkers, and well-designed clinical trials capable of clarifying the mechanisms, indications, and clinical value of regenerative interventions targeting the ovarian niche.

## 1 Introduction

Ovarian aging is a major determinant of female reproductive lifespan and represents one of the most complex forms of tissue aging in human physiology. Traditionally, this process has been viewed as the inevitable consequence of the progressive decline in the ovarian follicular reserve. However, accumulating evidence indicates that age-related reproductive deterioration is not exclusively driven by the quantitative loss of follicles but also by profound alterations affecting the ovarian microenvironment responsible for supporting follicular development and function (1–3).

Ovarian aging is now recognized as a multifactorial process involving stromal remodeling, fibrosis, vascular dysfunction, chronic low-grade inflammation, mitochondrial impairment, oxidative stress, and disruption of communication among the various cellular components of the ovary (2–4). These alterations contribute to the development of clinical conditions such as diminished ovarian reserve, poor ovarian response, and premature ovarian insufficiency, which represent an increasing challenge in reproductive medicine in the context of delayed childbearing observed across many societies (5, 6).

Historically, therapeutic strategies aimed at improving fertility have primarily focused on the follicle as the fundamental functional unit of the ovary. However, growing evidence suggests that follicular competence is highly dependent on the integrity of the ovarian niche that supports it. From this perspective, reproductive aging may be interpreted not only as a decline in follicular reserve but also as a progressive loss of the capacity of the ovarian microenvironment to sustain the survival, growth, and functional competence of the remaining follicles (1–3).

The clinical relevance of this issue is substantial. Although hormonal treatments may alleviate the endocrine consequences of ovarian insufficiency, they do not restore fertility (6). Likewise, assisted reproductive technologies remain largely dependent on the quantity and quality of residual follicles. For many women with premature ovarian insufficiency or severely compromised ovarian reserve, oocyte donation currently offers the highest probability of achieving pregnancy, although it may not be acceptable from a personal, genetic, or emotional perspective (7). This situation has stimulated the search for novel approaches capable of partially restoring residual ovarian function.

Within this context, regenerative medicine has emerged as an area of growing interest. Early investigations focused on the possibility of using stem cells to directly replace damaged ovarian cells. However, advances in regenerative biology have progressively shifted this view. Current evidence suggests that many of the beneficial effects observed with cellular therapies are mediated through paracrine mechanisms capable of modulating angiogenesis, inflammation, tissue remodeling, and intercellular communication within the ovarian niche (8–10).

In parallel, platelet-rich plasma (PRP) has emerged as one of the most accessible biological strategies for promoting ovarian regenerative processes. The high concentration of growth factors, cytokines, and bioactive mediators contained within platelets provides a biologically plausible basis for influencing tissue repair mechanisms and functional restoration of the ovarian microenvironment (11–13). However, interpretation of the available evidence is complicated by an often-overlooked assumption: the notion that PRP represents a biologically homogeneous intervention. Variations in composition, platelet concentration, leukocyte content, and preparation methods suggest that products grouped under the term “PRP” may in fact represent biologically distinct entities, potentially contributing to the heterogeneity observed across clinical studies (14–16).

This conceptual evolution has stimulated interest in combined regenerative approaches capable of acting simultaneously on both local and systemic components of tissue repair. Among these, the integration of platelet-derived biological products with stem cell mobilization strategies is particularly attractive from a biological standpoint, as it combines local regenerative signaling with mechanisms that may enhance systemic reparative potential (17–19). Nevertheless, the biological basis of these approaches remains insufficiently explored, particularly regarding the interaction between stem cell mobilization, paracrine signaling, and restoration of the ovarian niche.

**Figure 1:**
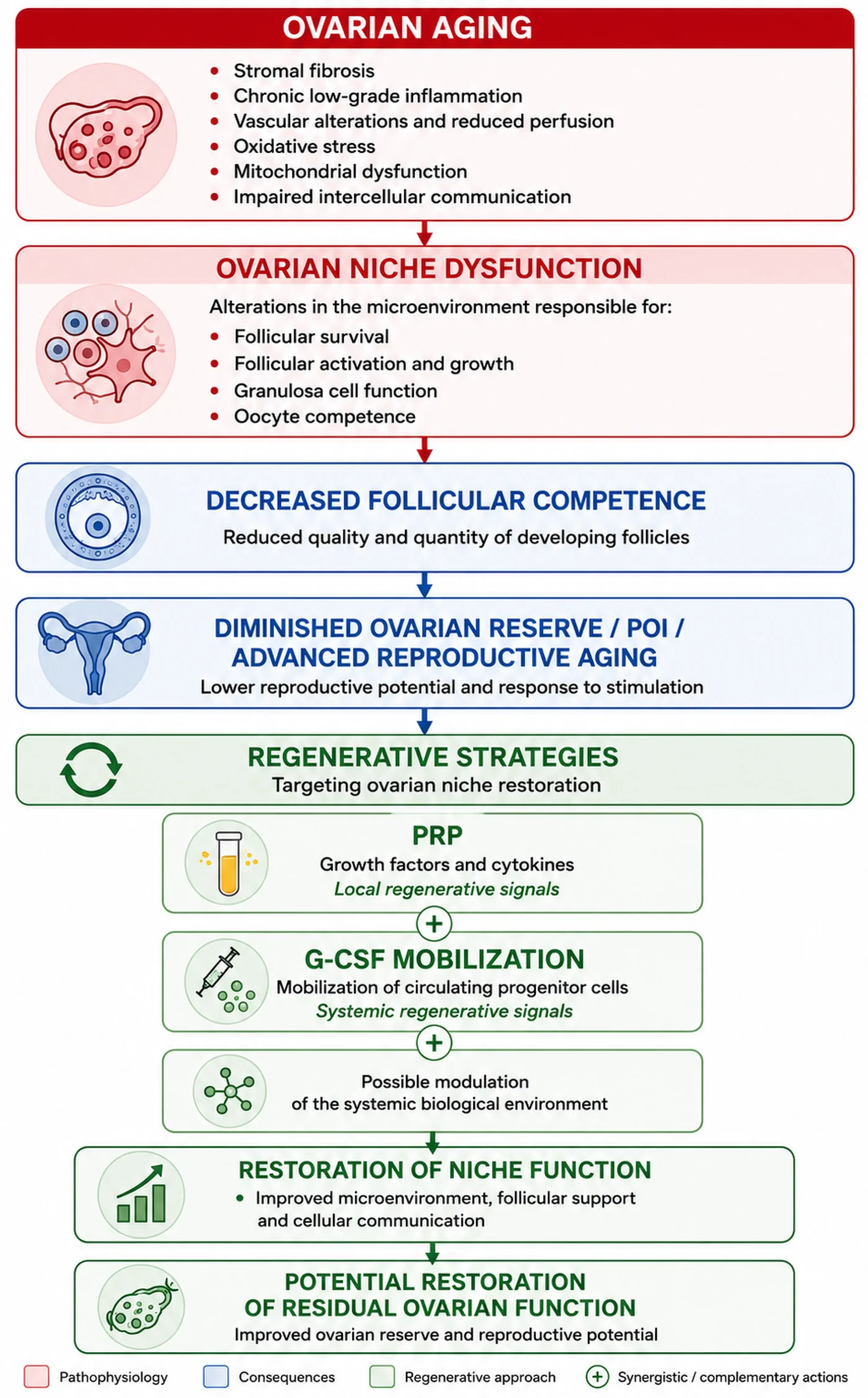
From the follicular paradigm to the ovarian niche paradigm: biological foundations of regenerative strategies for ovarian rejuvenation.

The aim of this review is to examine the biological rationale and clinical evidence supporting the use of PRP in reproductive medicine, to discuss the emerging role of the ovarian niche as a therapeutic target, and to explore the hypothesis that combining local and systemic regenerative signals may represent a promising strategy for restoring function in the aging or insufficient ovary. The transition from a follicle-centered paradigm to an ovarian niche-centered model and the rationale for regenerative interventions are summarized in Figure 1. Specifically, this review is built upon the hypothesis that restoration of ovarian niche functionality, rather than the isolated activation of residual follicles, may constitute the central biological objective of contemporary ovarian rejuvenation strategies.

## 2 Literature search strategy

This review was conducted as a structured narrative review aimed at critically summarizing the current evidence regarding platelet-rich plasma (PRP), ovarian niche restoration, and complementary regenerative strategies in reproductive medicine.

A structured literature search was performed using PubMed/MEDLINE, Scopus, and Web of Science. The search primarily included articles published between January 2000 and March 2026, although seminal earlier publications were incorporated when considered essential to provide biological or historical context. Additional relevant studies were identified through manual screening of the reference lists of selected articles.

The search strategy combined Medical Subject Headings (MeSH) and free-text terms related to the main topics addressed in this review, including *platelet-rich plasma*, *ovarian rejuvenation*, *ovarian aging*, *ovarian niche*, *poor ovarian response*, *premature ovarian insufficiency*, *ovarian fibrosis*, *regenerative medicine*, *mesenchymal stromal cells*, *stem cells*, *hematopoietic stem cell mobilization*, *granulocyte colony-stimulating factor*, *G-CSF*, *CXCL12*, *SDF-1*, and *CXCR4*. Additional searches were performed to identify randomized controlled trials, systematic reviews, meta-analyses, translational studies, and key mechanistic investigations relevant to ovarian regenerative therapies.

Publications were selected according to their scientific relevance, methodological quality, and contribution to the biological understanding or clinical application of ovarian regenerative strategies. Particular emphasis was placed on high-quality clinical evidence, including randomized controlled trials and systematic reviews, while observational studies, experimental investigations, and translational research were included when they provided important mechanistic insights or addressed emerging therapeutic concepts for which higher levels of evidence remain limited.

Given the narrative and hypothesis-generating nature of this review, no formal systematic review methodology or meta-analysis was undertaken. Instead, the available evidence was critically appraised and integrated to distinguish established clinical evidence from preclinical findings, mechanistic hypotheses, and future research perspectives, with the aim of providing a balanced overview of this rapidly evolving field.

Throughout the manuscript, special attention has been paid to differentiating evidence derived from randomized clinical trials, observational clinical studies, preclinical research, and biologically plausible hypotheses, thereby facilitating an evidence-based interpretation of current regenerative approaches in reproductive medicine.

**Figure 2:**
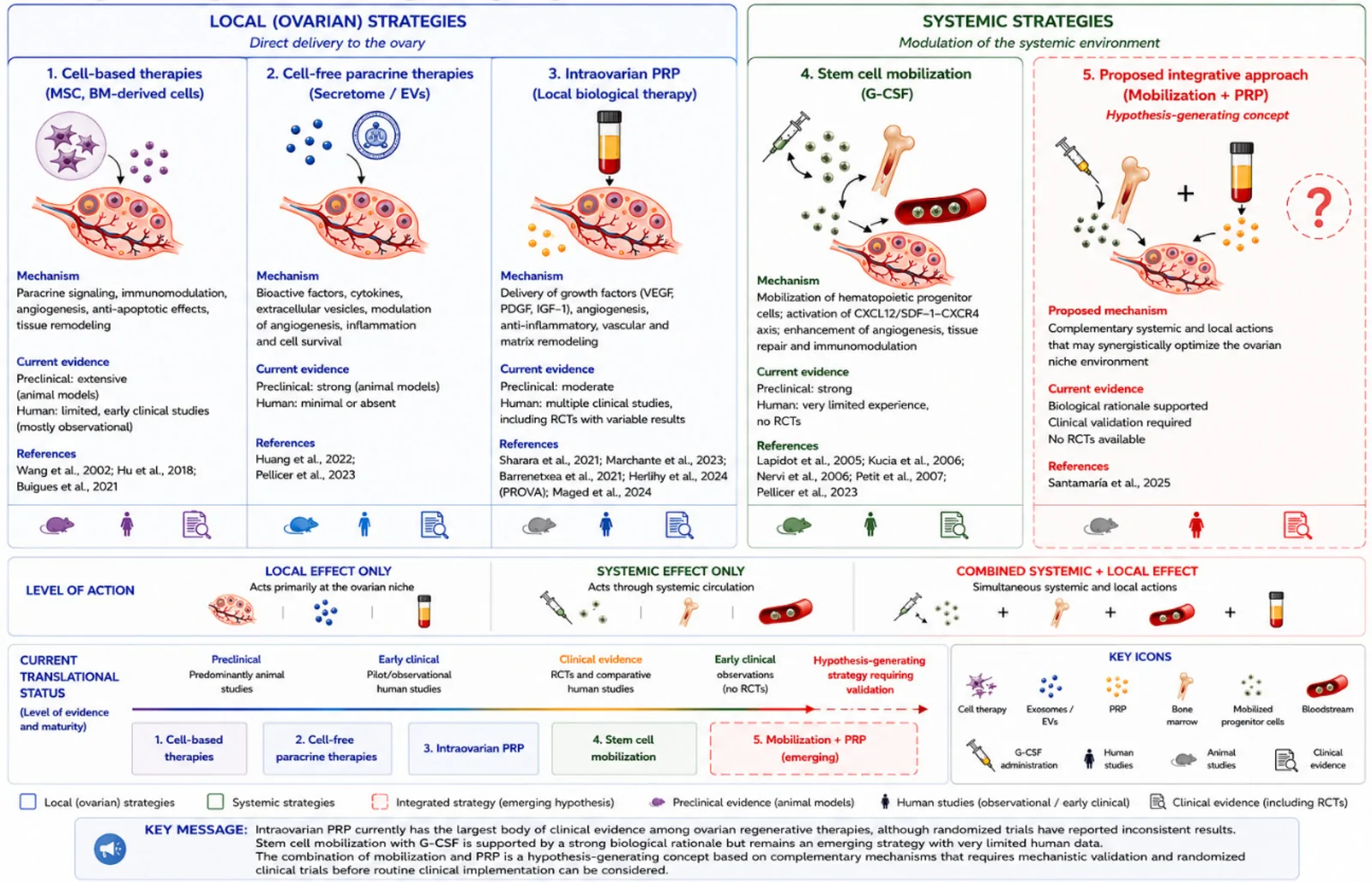
Regenerative strategies targeting ovarian niche restoration: current evidence and translational landscape.

This review was designed as a structured narrative review aimed at providing a balanced and critical appraisal of the available evidence. Consequently, although a transparent literature-search strategy was applied, the review does not constitute a formal systematic review or meta-analysis and therefore retains the inherent limitations of narrative evidence synthesis. The current regenerative strategies targeting ovarian niche restoration and their translational status are summarized in Figure 2.

## 3 Biological foundations of platelet-rich plasma

### 3.1 Composition of platelet-rich plasma

Platelet-rich plasma (PRP) is an autologous blood-derived concentrate obtained from peripheral blood through centrifugation procedures designed to increase platelet concentration above physiological levels. Although no universally accepted definition exists, most clinical PRP preparations contain approximately two- to five-fold higher platelet concentrations than baseline whole blood (11, 15).

From a biological perspective, PRP should be regarded as a complex platform of bioactive mediators rather than a simple suspension of platelets. In addition to plasma proteins and coagulation factors, PRP contains cytokines, chemokines, adhesion molecules, numerous growth factors stored within platelet alpha granules, and, depending on the preparation method, extracellular vesicles (11, 20). Following platelet activation, these components are released locally and generate signaling cascades capable of modulating tissue repair, cellular homeostasis, and regenerative processes (11, 13, 20).

The final composition of PRP is influenced by multiple technical variables, including centrifugation protocols, platelet concentration, leukocyte content, and activation methods. This biological heterogeneity represents one of the major limitations when comparing studies and likely contributes substantially to the variability of clinical outcomes reported across the literature (14, 15).

Several classification systems have been proposed to improve the biological characterization and reporting of PRP, including the PAW classification, the DEPA classification, and more recently the MARSPILL framework. Although none has achieved universal acceptance, these systems emphasize the importance of systematically reporting platelet concentration, leukocyte content, activation status, erythrocyte contamination, preparation protocols, and product purity. Adoption of standardized reporting frameworks will be essential for improving comparability among studies, facilitating interpretation of clinical outcomes, and advancing evidence-based development of ovarian regenerative therapies (14, 15).

This Table 1 summarizes the methodological heterogeneity in PRP preparation and reporting across representative clinical studies evaluating intraovarian PRP for ovarian rejuvenation or ovarian niche restoration. Variables include preparation system, centrifugation protocol, platelet concentration, leukocyte content, activation method, injected volume, number of injections, and treatment timing.

**Table 1:** Biological characterization and reporting quality of platelet-rich plasma (PRP) preparations in representative clinical studies.

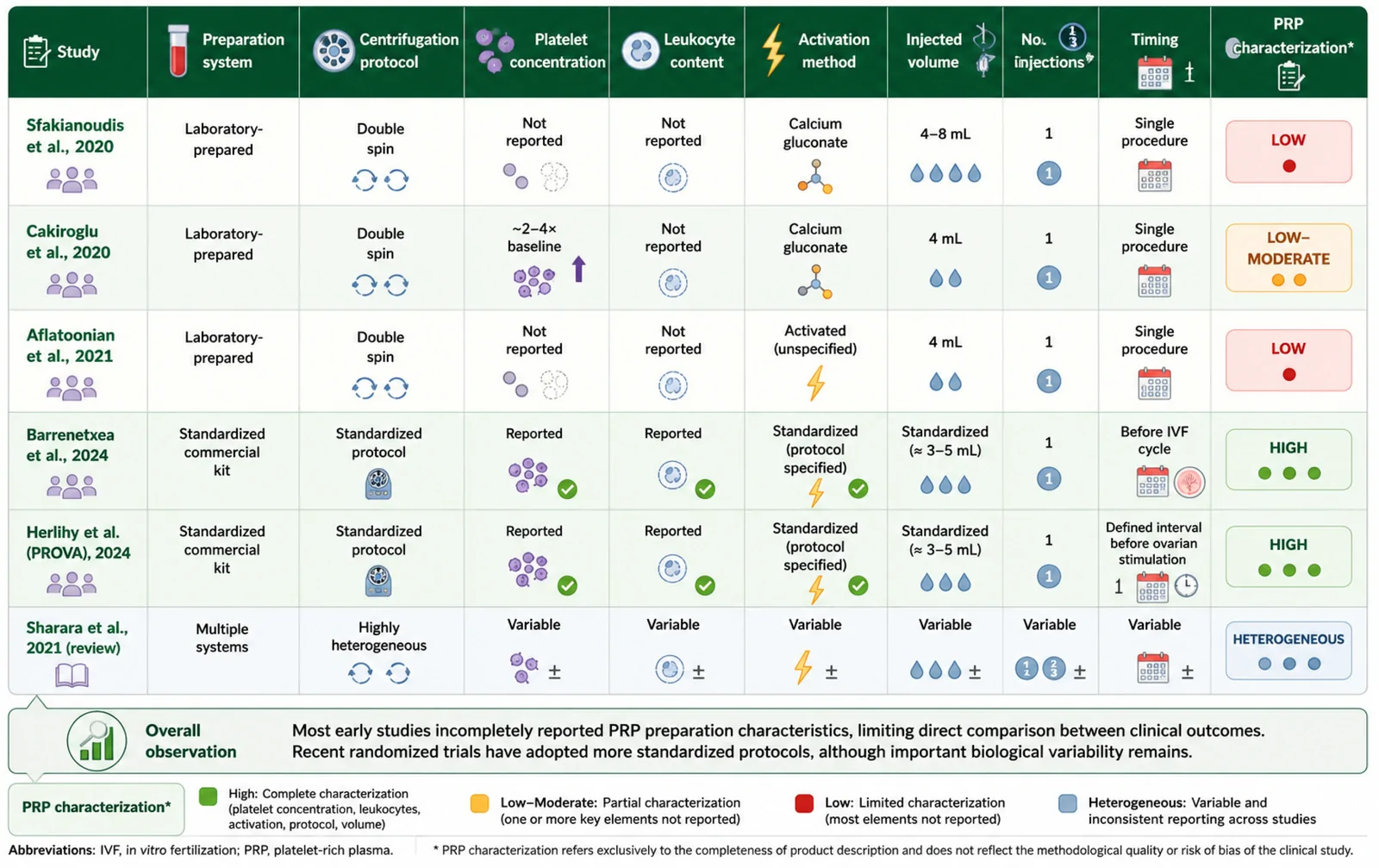

IVF, *in vitro* fertilization; PRP, platelet-rich plasma.

The final column reflects the completeness of PRP characterization reported in each study and refers exclusively to the description of the biological product (platelet concentration, leukocyte content, preparation protocol, activation method, and injected volume). It does not represent the methodological quality or risk of bias of the clinical study itself.

Overall, most early studies incompletely reported PRP preparation characteristics, limiting direct comparison between clinical outcomes. More recent randomized trials have adopted increasingly standardized protocols, although substantial biological heterogeneity remains across studies. This variability likely contributes to the inconsistent clinical outcomes reported in the current literature and highlights the need for standardized PRP preparation and reporting in future investigations (11, 21–25).

### 3.2 Growth factors and cytokines

The biological activity of PRP is primarily mediated by the coordinated release of growth factors and soluble mediators capable of regulating cell survival, proliferation, migration, and tissue repair. Among the most relevant factors are platelet-derived growth factor (PDGF), transforming growth factor-beta (TGF-*β*), vascular endothelial growth factor (VEGF), epidermal growth factor (EGF), insulin-like growth factor-1 (IGF-1), fibroblast growth factor (FGF), and hepatocyte growth factor (HGF) (11, 13, 20).

These mediators act in a coordinated manner on multiple cellular populations, promoting angiogenesis, stromal repair, extracellular matrix remodeling, and maintenance of cellular viability. In addition, PRP contains cytokines and chemokines with immunomodulatory and chemotactic properties that may contribute to intercellular communication and potentially participate in the recruitment of circulating progenitor cells (13, 26, 27).

The growing recognition of paracrine signaling as a central mechanism of tissue regeneration has brought PRP conceptually closer to stem cell–based therapies. In both settings, a substantial proportion of the biological effects appears to be mediated by soluble factors capable of modifying the tissue microenvironment rather than by direct cellular replacement (9, 28).

### 3.3 Effects on the ovarian microenvironment

The potential relevance of PRP in reproductive medicine lies in its capacity to simultaneously influence several mechanisms involved in ovarian niche deterioration. Multiple platelet-derived factors participate in angiogenic processes, promoting tissue perfusion and the local delivery of oxygen and nutrients, both of which are essential for maintaining follicular function (1, 3, 11, 13).

PRP may also modulate local inflammatory responses and contribute to extracellular matrix reorganization, processes that are particularly relevant in the aging ovary, which is characterized by stromal fibrosis, chronic low-grade inflammation, and impaired cellular communication (3, 13, 18, 29).

At the molecular level, numerous PRP-derived factors have been associated with the activation of intracellular pathways involved in cell survival, metabolism, and tissue repair, including PI3K/AKT and MAPK/ERK signaling pathways. These pathways have also been implicated in the biological effects observed with cellular therapies and stem cell–derived products (8, 11, 30).

Taken together, the available evidence suggests that the principal biological interest of PRP lies not in a direct effect on the follicle itself, but rather in its capacity to modify the microenvironment that supports residual ovarian function. This interpretation is consistent with the ongoing conceptual shift from follicle-centered approaches toward a model focused on restoration of ovarian niche functionality and provides a biological framework for understanding both the clinical findings reported with PRP and the growing interest in combined regenerative strategies (3, 18, 29, 31).

## 4 Biological basis of ovarian rejuvenation

### 4.1 Ovarian aging

Ovarian aging is a complex and multifactorial biological process that largely determines female reproductive lifespan. Traditionally, this phenomenon has been attributed to the progressive and irreversible depletion of the follicular reserve, which begins during fetal life and continues throughout the reproductive years. However, accumulating evidence over the past decade indicates that ovarian functional decline cannot be explained solely by a reduction in follicle number but also involves profound alterations in the tissue environment that supports follicular development and maturation (1–3, 31).

Key mechanisms implicated in ovarian aging include increased oxidative stress, accumulation of mitochondrial damage, genomic instability, epigenetic alterations, and dysregulation of metabolic pathways involved in cellular survival and energy homeostasis (3, 28). These changes affect not only oocytes but also granulosa cells, theca cells, stromal components, and the ovarian vascular network, progressively impairing the capacity of the ovary to sustain efficient folliculogenesis (3, 31).

Reduced tissue vascularization represents another hallmark of ovarian aging. Several studies have demonstrated a progressive decline in vascular density and local oxygen and nutrient supply, generating a microenvironment that is less favorable for follicular survival and oocyte quality (1, 2). In parallel, chronic low-grade inflammation associated with aging, commonly referred to as inflammaging, promotes stromal fibrosis and disrupts the cellular communication required for maintenance of reproductive function (2, 3).

Collectively, these findings have transformed the traditional view of ovarian aging as a purely quantitative phenomenon and have stimulated the development of regenerative approaches aimed at restoring ovarian tissue functionality beyond simply increasing the number of available follicles.

### 4.2 The ovarian niche

The concept of the ovarian niche has gained increasing relevance in the understanding of reproductive physiology and pathology. Similar to other regenerative tissues, the niche can be defined as the specialized microenvironment that provides structural, metabolic, and molecular support to resident cells, thereby regulating their survival, differentiation, and functional competence (2).

Within the ovary, this niche comprises a complex network of cellular and extracellular components, including granulosa cells, theca cells, stromal fibroblasts, immune cells, blood vessels, extracellular matrix, and multiple signaling molecules. The dynamic interaction among these components is essential for coordinating follicular activation, oocyte maturation, and ovarian endocrine function (2, 31).

The biological importance of the ovarian niche has become particularly evident through experimental studies in regenerative medicine. Several animal models have demonstrated that partial restoration of the ovarian microenvironment can improve follicular survival and recover reproductive function even in the absence of *de novo* follicle formation (18, 29). These findings suggest that part of the age-related decline in ovarian function may result from potentially reversible alterations of the tissue environment rather than from an absolute and irreversible loss of ovarian functional potential.

From this perspective, ovarian rejuvenation may be viewed as a process aimed at restoring the biological conditions required for residual follicles to fully express their functional competence. This represents an important conceptual shift from early approaches focused primarily on cellular replacement and provides a coherent biological framework for understanding the potential effects of PRP and other regenerative interventions (18, 29, 31).

### 4.3 Resident stem cells and progenitor cells

One of the most debated topics in modern reproductive biology is the possible existence of cell populations with regenerative capacity within the adult ovary. For decades, the prevailing paradigm held that the female follicular reserve was finite and non-renewable after birth. However, several groups have described cellular populations exhibiting characteristics consistent with ovarian stem or progenitor cells, including the expression of markers associated with pluripotency and self-renewal (7, 8).

Although the capacity of these cells to generate functional oocytes in humans remains highly controversial, increasing evidence supports the presence of progenitor cell populations with reparative potential within ovarian tissue. These cells may participate in tissue maintenance, local repair, and modulation of the ovarian microenvironment through the secretion of bioactive factors (8, 9).

In parallel, numerous studies have demonstrated that bone marrow–derived progenitor cells can migrate toward damaged tissues in response to inflammatory or regenerative signals. Within the ovary, hematopoietic and mesenchymal progenitor cells have been proposed to contribute to tissue repair, angiogenesis, and stromal remodeling (17, 32). These mechanisms appear to depend largely on chemotactic signaling pathways capable of recruiting progenitor cells to injured tissues, with the CXCL12/SDF-1–CXCR4 axis representing one of the best-characterized systems in regenerative biology (26, 27, 33).

The growing understanding of these mechanisms has encouraged the development of therapeutic strategies aimed not only at administering exogenous cells but also at stimulating endogenous repair processes through the mobilization and recruitment of circulating progenitor cells (17, 26, 27, 32).

### 4.4 Current controversies

Despite the rapid expansion of the ovarian rejuvenation field, important scientific and clinical controversies remain. The first concerns the existence and physiological relevance of functional germline stem cells in the adult human ovary. Although several studies have described cell populations with progenitor-like characteristics, definitive evidence demonstrating their contribution to the generation of new oocytes remains insufficient and highly debated (7).

Another unresolved issue relates to the mechanisms underlying the improvements reported after regenerative interventions. Current evidence suggests that the observed clinical benefits are unlikely to result primarily from the direct differentiation of transplanted cells into functional ovarian structures. Instead, the prevailing hypothesis proposes that modulation of the ovarian niche through paracrine, angiogenic, and immunomodulatory signaling represents the principal mechanism of action of these therapies (8, 9).

Considerable methodological heterogeneity also persists among studies, including differences in patient selection, definitions of ovarian insufficiency, treatment protocols, response criteria, and outcome measures. This variability complicates comparisons across studies and limits the ability to draw definitive conclusions regarding the clinical efficacy of currently available regenerative strategies (7, 34).

Finally, debate continues regarding the true biological scope of interventions currently grouped under the concept of ovarian rejuvenation. Although several studies have reported improvements in hormonal parameters, follicular activity, and reproductive outcomes, it remains unclear whether these findings reflect genuine restoration of ovarian niche functionality or merely the transient activation of previously quiescent residual follicles. This distinction carries important biological and clinical implications, as temporary recovery of follicular activity does not necessarily imply a durable modification of the mechanisms responsible for ovarian aging. Determining the extent to which regenerative interventions are capable of reversing the structural and functional alterations of the ovarian niche remains one of the central challenges of the field and strongly influences the interpretation of much of the currently available clinical evidence (7, 13, 35).

Ovarian aging is associated with stromal fibrosis, chronic low-grade inflammation, vascular dysfunction, oxidative stress, and impaired intercellular communication, all of which contribute to the progressive deterioration of the ovarian niche. From this perspective, the decline in follicular competence may be interpreted as a consequence of alterations in the microenvironment responsible for sustaining residual ovarian function. Contemporary regenerative approaches aim to target this niche through locally delivered signals provided by platelet-rich plasma (PRP) and systemic regenerative signals derived from stem cell mobilization strategies, with the goal of restoring ovarian microenvironment functionality and preserving or recovering residual reproductive potential (8–11, 13, 15, 17–19, 29).

## 5 Clinical evidence of Prp in reproductive medicine

Clinical investigation of intraovarian platelet-rich plasma (PRP) has expanded considerably during the last decade, reflecting the growing interest in regenerative approaches aimed at restoring ovarian function. Initial observational studies reported encouraging improvements in ovarian reserve markers, follicular development, and, in some cases, reproductive outcomes following PRP administration, generating considerable enthusiasm regarding its potential clinical application (11, 21–23).

However, interpretation of these early findings requires caution. Most published studies are characterized by substantial methodological heterogeneity, including differences in patient selection, clinical indications, PRP preparation protocols, platelet concentration, leukocyte content, activation methods, injected volume, treatment timing, outcome measures, and duration of follow-up. Moreover, many studies were observational, involved relatively small patient cohorts, and lacked appropriate control groups, thereby limiting the strength of the available clinical evidence (11, 16, 36).

More recently, randomized controlled trials have provided a more rigorous evaluation of intraovarian PRP. Importantly, these studies have generally reported more modest effects than those suggested by earlier observational investigations. The randomized placebo-controlled PROVA trial failed to demonstrate significant improvements in clinically meaningful reproductive outcomes following intraovarian PRP administration, whereas the randomized study by Barrenetxea et al. observed improvements in selected intermediate parameters, such as mature oocyte yield, without demonstrating consistent benefits in pregnancy or live birth outcomes. Collectively, these findings indicate that any biological effect of PRP, if present, is likely to be modest and may depend on careful patient selection, standardized PRP preparation protocols, and appropriate timing of treatment (24, 25).

Consequently, although PRP remains one of the most extensively investigated regenerative strategies in reproductive medicine, current randomized evidence does not support the routine clinical implementation of intraovarian PRP. Nevertheless, the absence of demonstrated efficacy should not be interpreted as evidence of biological ineffectiveness, given the substantial heterogeneity in PRP formulations, patient populations, and study methodologies currently available. Future clinical studies should prioritize standardized biological characterization of PRP products, harmonized clinical protocols, adequately powered randomized controlled trials, and clinically meaningful reproductive endpoints in order to determine which patients, if any, are most likely to benefit from this intervention (16, 36).

This Table 2 summarizes the principal clinical studies investigating intraovarian PRP in women with diminished ovarian reserve (DOR), premature ovarian insufficiency (POI), poor ovarian response (POR), and advanced reproductive aging. For each study, the design, study population, sample size, PRP protocol, primary outcomes, principal findings, and overall conclusions are presented to facilitate comparison of the currently available clinical evidence.

**Table 2:** Summary of the principal clinical studies evaluating intraovarian platelet-rich plasma (PRP) for ovarian regenerative therapies.

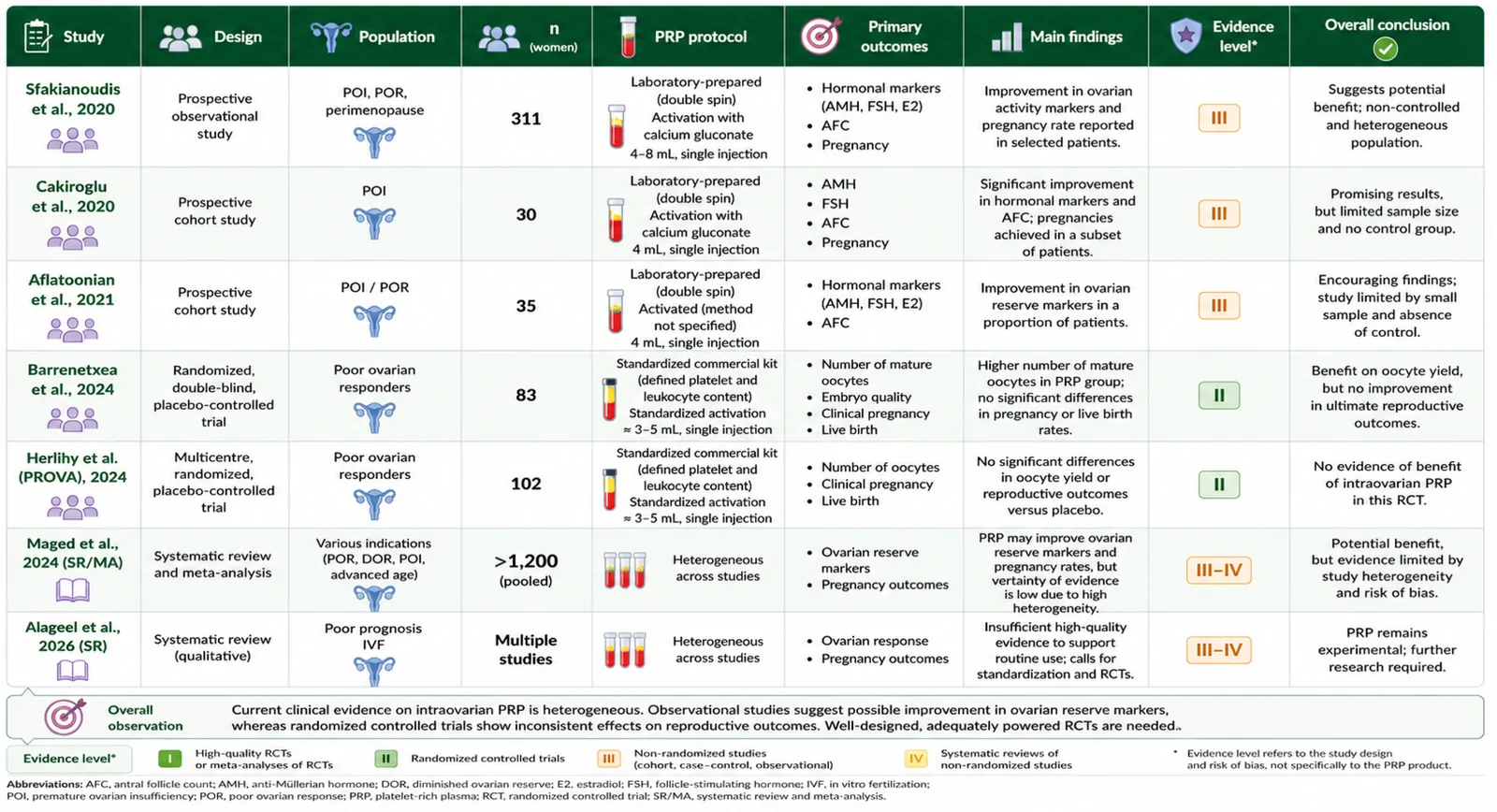

**Evidence level** refers to the hierarchy of study design and should not be interpreted as a measure of treatment efficacy.

AFC, antral follicle count; AMH, anti-Müllerian hormone; DOR, diminished ovarian reserve; E2, estradiol; FSH, follicle-stimulating hormone; IVF, *in vitro* fertilization; POI, premature ovarian insufficiency; POR, poor ovarian response; PRP, platelet-rich plasma; RCT, randomized controlled trial.

The evidence illustrates substantial methodological heterogeneity across studies, including differences in patient selection, PRP preparation protocols, clinical endpoints, follow-up duration, and outcome reporting. While several observational studies have reported improvements in ovarian reserve markers or reproductive outcomes, randomized controlled trials have yielded inconsistent or largely negative results regarding clinically meaningful reproductive endpoints, highlighting the need for cautious interpretation.

Overall, the current evidence suggests that intraovarian PRP remains an investigational regenerative approach. Although preliminary findings are encouraging in selected patient populations, routine clinical implementation cannot yet be recommended owing to the limited number of adequately powered randomized controlled trials, variability in PRP characterization, and the absence of standardized treatment protocols.

Importantly, the progressive improvement in study design observed over time has been accompanied by increasingly rigorous evaluation of PRP products, underscoring the critical need for standardized biological characterization and adequately powered randomized clinical trials to determine the true therapeutic value of ovarian regenerative interventions (16, 21–25, 36).

### 5.1 Diminished ovarian reserve

Diminished ovarian reserve represents one of the most frequently studied indications for intraovarian PRP administration. In this setting, the therapeutic objective is not necessarily to increase the absolute number of available follicles, but rather to optimize the function of those that remain active within a progressively deteriorating ovarian microenvironment (5, 37).

Several observational studies have reported increases in anti-Müllerian hormone (AMH) levels, reductions in follicle-stimulating hormone (FSH), increases in antral follicle count, and improved ovarian response to stimulation following PRP administration (21–23). These findings are biologically plausible and consistent with the angiogenic, immunomodulatory, and regenerative effects attributed to platelet-derived growth factors and bioactive mediators (11–13).

However, more recent data from controlled studies have yielded less conclusive results. The trial by Barrenetxea et al. reported an increase in the cumulative number of mature oocytes retrieved after treatment but did not demonstrate consistent benefits in more advanced reproductive outcomes such as embryo quality, euploidy rates, or clinical pregnancy (24). Similarly, the PROVA trial conducted by Herlihy et al. failed to demonstrate clinically meaningful improvements in the principal reproductive endpoints evaluated (25).

These findings should nevertheless be interpreted with caution. Both studies represent important methodological advances compared with much of the earlier literature, yet several factors may have influenced their ability to detect clinically meaningful biological effects. These include differences in PRP preparation and characterization, limited statistical power for complex reproductive outcomes, and uncertainty regarding the optimal interval required for tissue remodeling processes induced by PRP to translate into measurable clinical changes.

Consequently, although favorable signals have been observed in intermediate markers of ovarian function, the true magnitude of the clinical benefit remains to be fully established.

### 5.2 Premature ovarian insufficiency

Premature ovarian insufficiency (POI) represents one of the most compelling clinical settings for regenerative medicine because of the limited availability of therapeutic options capable of restoring residual ovarian function (7).

Early clinical experiences with intraovarian PRP described partial recovery of ovarian activity, resumption of menstrual cycles, hormonal improvements, and spontaneous or assisted pregnancies in a subset of women previously considered to have a very poor reproductive prognosis (21, 22).

Interpretation of these findings remains challenging because of the biological heterogeneity of the condition itself. POI does not necessarily imply complete absence of follicular activity, and some women may experience spontaneous episodes of ovarian recovery. Furthermore, most available studies lack adequate control groups and are limited by small sample sizes (7, 35).

Nevertheless, recurrent reports of favorable outcomes across different cohorts and clinical series have sustained scientific interest in this approach and stimulated the development of novel strategies aimed at enhancing the observed regenerative effects (16).

### 5.3 Women of advanced reproductive Age

Women of advanced reproductive age constitute another population of increasing interest for ovarian rejuvenation strategies. In these patients, aging simultaneously affects follicular reserve, oocyte quality, and ovarian niche functionality (1, 3, 31).

From a biological perspective, PRP may exert its effects primarily through optimization of the tissue environment in which residual follicles develop, promoting processes related to angiogenesis, cellular metabolism, and intercellular communication. Several studies have reported improvements in ovarian responsiveness and selected reproductive outcomes, although the magnitude of these effects varies considerably across cohorts and treatment protocols (11, 13).

Current evidence does not support the conclusion that PRP reverses the fundamental mechanisms of ovarian aging. However, it suggests that certain patients may benefit from a transient improvement in the biological environment required for residual follicular function, a hypothesis consistent with experimental models emphasizing restoration of ovarian niche functionality as a key mechanism underlying reproductive recovery (18, 29).

### 5.4 Clinical outcomes and methodological limitations

The available clinical evidence suggests that intraovarian PRP may induce favorable changes in selected markers of ovarian function and, in some cases, improve ovarian responsiveness and reproductive outcomes. Nevertheless, the overall quality of the evidence remains limited and heterogeneous (16, 35, 36).

Among the major limitations are the lack of standardization of PRP protocols, variability in platelet concentration, differences in activation methods, heterogeneity of study populations, and the use of outcome measures that are not always comparable across studies (15, 38).

The recent emergence of randomized controlled trials has represented an important step forward in the evaluation of intraovarian PRP and has contributed to raising the methodological quality of the field. In particular, the studies by Barrenetxea et al. and Herlihy et al. have provided valuable data derived from more rigorous designs than those employed in much of the earlier literature (24, 25). However, their findings have not consistently confirmed the improvements reported in numerous observational studies, generating considerable debate regarding the true magnitude of treatment-related benefits.

These results should be interpreted within the context of a field that remains in active development. Variables such as PRP preparation and characterization, platelet concentration, the potential biological effects of ovarian puncture itself, patient selection, endpoint definition, and the time required for tissue remodeling processes remain insufficiently standardized. In addition, complex reproductive outcomes (including embryo quality, euploidy, and live birth), require substantially larger sample sizes to detect clinically meaningful differences.

An often-underappreciated conceptual limitation in the interpretation of the available literature is the assumption that PRP represents a biologically homogeneous intervention. In reality, preparations grouped under the term “PRP” may differ substantially in platelet concentration, leukocyte content, growth factor profile, activation methods, and biological context of collection. Consequently, studies that appear to evaluate the same intervention may in fact be assessing biologically distinct products with different regenerative properties. This heterogeneity may contribute substantially to the variability observed across clinical studies and may help explain some of the discrepancies among observational studies, randomized trials, and systematic reviews (14–16).

From this perspective, apparently conflicting results should not necessarily be interpreted as evidence for or against PRP itself, but rather as an indication that the biological conditions under which these interventions may be effective remain incompletely understood. Rather than challenging the biological plausibility of the approach, recent trials highlight the need to define more precisely which formulations, treatment protocols, and patient populations are most likely to benefit from regenerative therapies (13). This observation becomes particularly relevant in light of emerging strategies that combine PRP with stem cell mobilization and other regenerative approaches designed to act simultaneously on both local and systemic components of tissue repair.

Accordingly, the current challenge is no longer simply determining whether PRP works, but rather identifying which formulations, protocols, and patient **profiles** are most appropriate for harnessing its biological potential within a broader framework of ovarian regenerative medicine.

## 6 Hematopoietic stem cell mobilization: biological rationale and current evidence

The concept of ovarian regeneration has progressively evolved beyond the exclusive administration of local biological products toward strategies aimed at modifying both the ovarian microenvironment and the systemic regenerative milieu. Within this context, hematopoietic stem cell mobilization induced by granulocyte colony-stimulating factor (G-CSF) has emerged as a biologically plausible approach that may complement local regenerative therapies through mechanisms extending beyond the simple increase in circulating progenitor cells. Although clinical evidence remains limited, accumulating experimental and translational data provide a rationale for further investigation of this strategy (7, 26, 27, 33, 39).

Current regenerative approaches targeting ovarian niche restoration are organized according to their biological origin (local ovarian therapies versus systemic regenerative strategies), principal mechanism of action, level of biological intervention, current clinical evidence, and translational status. Cell-based therapies and cell-free paracrine approaches remain predominantly supported by preclinical research. Intraovarian platelet-rich plasma (PRP) currently represents the regenerative intervention with the largest body of human clinical evidence, although substantial methodological heterogeneity and inconsistent findings from randomized controlled trials indicate that routine clinical implementation cannot yet be recommended (8–11, 16, 18, 24, 25, 29).

Systemic hematopoietic stem cell mobilization induced by granulocyte colony-stimulating factor (G-CSF) is supported by a well-established biological rationale involving activation of the CXCL12/SDF-1–CXCR4 signaling axis, angiogenesis, immunomodulation, vascular remodeling, and tissue repair. However, its application in ovarian regenerative medicine is currently supported by only limited early clinical experience, and randomized controlled trials are not yet available (7, 26, 27, 33, 39).

The proposed integration of G-CSF-induced hematopoietic stem cell mobilization with intraovarian PRP is presented as an **emerging translational hypothesis**, based on complementary systemic and local regenerative mechanisms rather than demonstrated therapeutic efficacy. To date, no randomized clinical trials have evaluated this combined approach, no biological characterization of PRP collected following G-CSF-induced mobilization has been reported, and no dose–response relationship between circulating CD34+ cell mobilization and ovarian outcomes has been demonstrated. Consequently, the proposed integrative strategy should be regarded as an investigational translational concept requiring mechanistic validation together with adequately powered randomized clinical trials before routine clinical implementation can be considered (16, 19, 24, 25).

The lower panels summarize the predominant level of biological action, the current stage of translational development, and the relative strength of the available evidence supporting each regenerative strategy. The translational status illustrated in the figure reflects the current level of scientific evidence and should not be interpreted as a ranking of therapeutic efficacy or clinical recommendation.

Overall, the figure illustrates the transition of ovarian regenerative medicine from biologically plausible concepts toward evidence-based clinical translation, highlighting both current achievements and the principal knowledge gaps that should guide future mechanistic and clinical research.

### 6.1 Biological rationale

Hematopoietic stem cell mobilization induced by G-CSF results in the transient release of CD34+ progenitor cells and other bone marrow-derived cell populations into the peripheral circulation. This process involves coordinated regulation of the CXCL12/SDF-1–CXCR4 signaling axis, one of the principal mechanisms controlling stem cell trafficking, tissue homing, angiogenesis, and regenerative responses following tissue injury (26, 27, 33).

Beyond increasing the availability of circulating progenitor cells, G-CSF induces complex systemic biological changes that include cytokine modulation, immune regulation, extracellular matrix remodeling, endothelial activation, and enhanced angiogenic signaling. Collectively, these processes may create a transient systemic regenerative environment potentially facilitating tissue repair in multiple organs (39).

**Figure 3:**
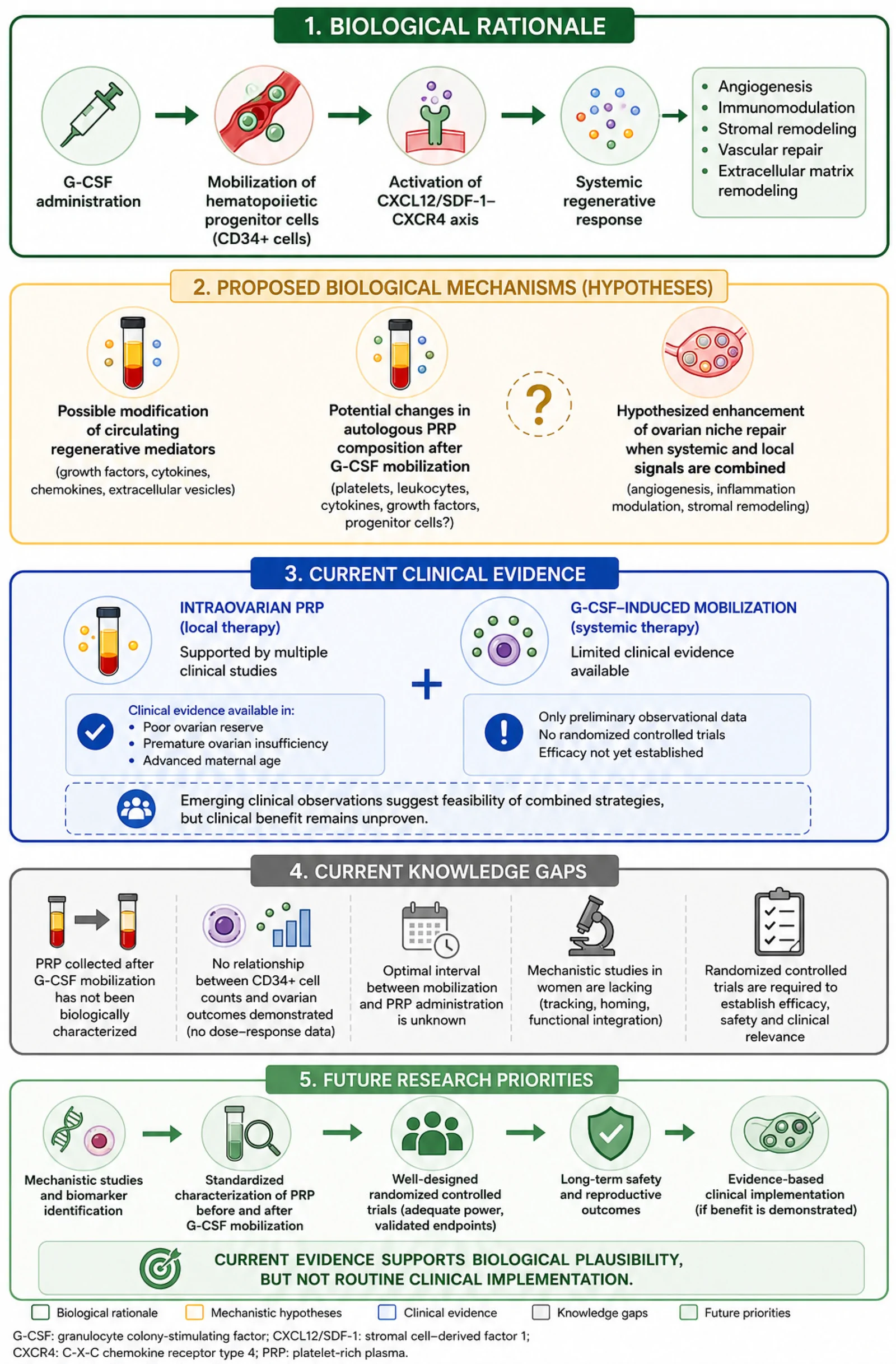
Biological rationale and current translational status of G-CSF-induced hematopoietic stem cell mobilization in ovarian regenerative medicine.

Within the ovary, these mechanisms are biologically relevant because ovarian aging is increasingly recognized as a consequence not only of progressive follicular depletion but also of deterioration of the surrounding stromal and vascular microenvironment. Restoration of angiogenesis, reduction of chronic inflammation, extracellular matrix remodeling, and improvement of cellular communication within the ovarian niche therefore represent plausible biological targets for regenerative interventions (1–3). The biological rationale and current translational status of G-CSF-induced stem cell mobilization are summarized in Figure 3.

### 6.2 Preclinical and translational evidence

Experimental studies investigating stem cell mobilization and regenerative medicine have demonstrated that bone marrow-derived cells participate in tissue repair predominantly through paracrine mechanisms rather than long-term engraftment or transdifferentiation. Secreted cytokines, extracellular vesicles, growth factors, and immunomodulatory mediators appear to account for most of the observed regenerative effects (8–10).

Similarly, increasing evidence suggests that PRP exerts its biological activity primarily through modulation of the local microenvironment rather than direct replacement of damaged cells. Consequently, both approaches may share complementary mechanisms involving angiogenesis, stromal remodeling, inflammatory regulation, and restoration of tissue homeostasis (7, 29, 40).

This conceptual overlap may provide the biological rationale for investigating whether systemic stem cell mobilization could potentiate the regenerative effects of locally administered PRP. However, this hypothesis remains largely theoretical and has not yet been validated by mechanistic human studies.

### 6.3 Current clinical evidence

Clinical evidence evaluating hematopoietic stem cell mobilization in ovarian regenerative medicine remains scarce. To date, no adequately powered randomized controlled trials have assessed the efficacy of combining G-CSF-induced stem cell mobilization with intraovarian PRP administration. The currently available human evidence therefore consists primarily of preliminary observational studies and early translational experience.

Santamaría et al. (19) reported encouraging preliminary clinical observations using G-CSF-induced hematopoietic stem cell mobilization combined with intraovarian PRP in women with severe ovarian dysfunction. Although these preliminary findings from our group are encouraging, they derive from an observational, non-controlled cohort and were not designed to establish clinical efficacy. Thus, they should be interpreted as hypothesis-generating rather than definitive validation of the proposed therapeutic strategy, highlighting the need for independent, adequately powered randomized controlled trials (19).

Importantly, the biological composition of PRP obtained following hematopoietic stem cell mobilization was not characterized in the available clinical study. Therefore, it remains unknown whether G-CSF administration modifies the cellular or molecular composition of subsequently prepared autologous PRP, including platelet-derived growth factors, cytokines, extracellular vesicles, or circulating progenitor cell content. Whether any potential therapeutic effect results from systemic stem cell mobilization itself, altered PRP composition, or the interaction between both mechanisms remains entirely speculative.

While hematopoietic stem cell transplantation relies on well-defined CD34+ cell dose thresholds to achieve durable systemic engraftment, ovarian regenerative therapies are more likely to depend on localized paracrine signaling rather than long-term cellular engraftment. Consequently, extrapolating hematological dose-response paradigms to reproductive regenerative medicine is currently not justified, and the relationship between mobilized CD34+ cells and ovarian outcomes remains to be established.

### 6.4 Safety, regulatory considerations and future research priorities

Although both intraovarian PRP and G-CSF administration have generally demonstrated acceptable short-term safety profiles in their respective clinical settings, important uncertainties remain regarding their application as regenerative therapies for ovarian dysfunction. Current clinical studies are limited by relatively small sample sizes, heterogeneous treatment protocols, and short follow-up periods, precluding definitive conclusions regarding long-term safety or sustained clinical efficacy (11, 16, 25).

Although theoretical concerns regarding long-term hematopoietic consequences, including clonal hematopoiesis, have occasionally been raised in the context of G-CSF administration, these considerations should be interpreted in light of the extensive safety experience accumulated in healthy stem cell donors. Large prospective registries from the National Marrow Donor Program (NMDP) and the European Society for Blood and Marrow Transplantation (EBMT), comprising tens of thousands of healthy donors exposed to G-CSF, have not demonstrated an increased incidence of hematological malignancies or sustained clonal hematopoietic disorders compared with the general population (41–43). This extensive body of evidence supports the favorable long-term safety profile of G-CSF in healthy donors and provides important reassurance when considering its potential application in regenerative medicine. Nevertheless, because ovarian regenerative applications differ substantially from hematopoietic transplantation in both objectives and patient populations, dedicated long-term reproductive safety studies remain warranted.

Likewise, the reproductive implications of repeated regenerative interventions remain largely unknown. Future investigations should incorporate long-term follow-up evaluating ovarian function, reproductive outcomes, obstetric safety, offspring health, and potential late adverse events. Standardized safety reporting should become an essential component of future randomized clinical trials.

From a translational perspective, future studies should also prioritize standardized biological characterization of PRP products, harmonized preparation protocols, validation of predictive biomarkers, mechanistic evaluation of PRP obtained before and after G-CSF-induced mobilization, and determination of whether the magnitude of CD34+ mobilization correlates with clinical outcomes. These studies will be essential to clarify the biological mechanisms underlying ovarian regenerative therapies and to identify patient populations most likely to benefit.

Accordingly, although the biological rationale supporting integrated regenerative strategies is compelling, current evidence remains insufficient to justify routine clinical implementation outside appropriately designed clinical research protocols.

G-CSF administration induces transient mobilization of hematopoietic progenitor cells through activation of the CXCL12/SDF-1–CXCR4 signaling axis, resulting in systemic regenerative responses including angiogenesis, immunomodulation, vascular repair, and extracellular matrix remodeling (26, 27, 33, 39).

Based on these biological mechanisms, it has been hypothesized that systemic stem cell mobilization could complement the local regenerative effects of intraovarian platelet-rich plasma (PRP). However, the biological interaction between both interventions remains incompletely understood.

Importantly, no study has yet characterized the biological composition of PRP obtained following G-CSF-induced mobilization, and no relationship between circulating CD34+ cell counts and ovarian outcomes has been demonstrated. Consequently, the proposed interaction between systemic mobilization and local PRP administration should currently be regarded as a biologically plausible hypothesis rather than an established therapeutic mechanism.

Future translational studies comparing PRP prepared before and after G-CSF administration, together with adequately powered randomized clinical trials, will be essential to determine whether combined regenerative strategies provide clinically meaningful benefit beyond PRP alone (7, 11, 16, 19, 24–27).

## 7 Future perspectives and challenges

### 7.1 Standardization of PRP

One of the major obstacles to interpreting the available clinical evidence regarding PRP in reproductive medicine is the substantial heterogeneity of preparation and administration protocols. At present, no universal consensus exists regarding optimal platelet concentration, leukocyte content, activation methods, administered volume, or treatment frequency, making comparisons across studies and reproducibility of findings particularly challenging (14, 15).

Variability in the biological composition of PRP may translate into substantial differences in the concentration of growth factors, cytokines, and inflammatory mediators, resulting in products with potentially distinct functional profiles. Consequently, studies evaluating interventions generically labeled as “PRP” may in fact be investigating biologically different products (15).

Standardization of collection, characterization, and administration protocols therefore represents a priority for the future development of the field. Systematic incorporation of quality-control parameters, including platelet count, cellular composition, and molecular profiling of PRP preparations, may improve comparability among studies and facilitate identification of formulations with greater therapeutic potential.

Furthermore, the growing interest in secretome-enriched biological products and combined strategies involving cell mobilization reinforces the need for more precise characterization criteria capable of clarifying the relationship between biological composition and clinical response.

### 7.2 Biomarkers

The identification of biomarkers capable of predicting treatment response represents another major challenge for ovarian rejuvenation strategies. The substantial heterogeneity observed among patients suggests that the potential efficacy of these interventions is likely influenced by individual biological factors that remain incompletely characterized (5, 37).

To date, most studies have relied on conventional parameters such as serum anti-Müllerian hormone (AMH), follicle-stimulating hormone (FSH), and antral follicle count to evaluate ovarian response. Although these variables remain essential clinical tools, they possess important limitations in reflecting the complex biological processes involved in ovarian niche regeneration (5).

The development of more sophisticated biomarkers could improve patient selection and provide deeper insight into the mechanisms underlying regenerative interventions. Potential candidates include markers related to angiogenesis, inflammation, oxidative stress, tissue remodeling, and chemotactic signaling, including components associated with the CXCL12/SDF-1–CXCR4 axis and progenitor cell mobilization (26, 27).

In the future, integration of molecular, cellular, and imaging biomarkers may facilitate a more personalized approach to ovarian rejuvenation, helping identify patients who retain a biologically recoverable ovarian niche and distinguishing them from those with more advanced tissue deterioration.

A particularly relevant question for future investigation is whether hematopoietic mobilization merely increases the availability of circulating progenitor cells or also induces quantitative and qualitative changes in the biological composition of autologous products subsequently obtained from peripheral blood. Characterization of potential variations in growth factors, cytokines, chemokines, and other regenerative mediators may help explain part of the variability observed among different therapeutic strategies. Moreover, identification of these changes could facilitate the development of functional biomarkers associated with individual regenerative capacity and improve selection of candidates for therapies combining cell mobilization and regenerative biological products.

### 7.3 Controlled clinical trials

Despite the increasing number of publications addressing PRP and ovarian regenerative medicine, the overall methodological quality of the available clinical evidence remains limited. Most published studies consist of case series, observational cohorts, or investigations with relatively small sample sizes, restricting the ability to draw definitive conclusions regarding clinical efficacy (16, 35, 36).

In addition to heterogeneity in treatment protocols, important differences persist in diagnostic criteria, patient selection, clinical endpoints, and follow-up duration. These factors limit direct comparisons among studies and contribute to the persistence of apparently conflicting findings (13, 14).

Future clinical trials should incorporate randomized controlled designs, adequate sample sizes, and clearly defined inclusion criteria. Equally important will be the establishment of clinically meaningful endpoints extending beyond hormonal and ultrasonographic changes to include clinical pregnancy, live birth, and long-term reproductive outcomes.

Uncertainty also remains regarding the most appropriate endpoints for evaluating ovarian rejuvenation strategies. Although hormonal and imaging parameters provide valuable information regarding ovarian activity, their relationship with clinically meaningful reproductive outcomes remains a matter of debate.

However, conducting controlled trials in this field presents specific challenges. Women with premature ovarian insufficiency or severely diminished ovarian reserve represent a particularly vulnerable reproductive population, often with limited time available to pursue pregnancy. Under these circumstances, assignment to placebo or control groups may significantly hinder recruitment and increase study attrition, potentially affecting both feasibility and statistical power.

For this reason, in addition to randomized controlled trials, evidence derived from real-world evidence (RWE) studies may play an important complementary role in knowledge generation. Although such studies possess inherent limitations related to the absence of randomization and the potential influence of confounding factors, they allow evaluation of interventions under routine clinical conditions, inclusion of more heterogeneous patient populations, and generation of clinically relevant hypotheses for future investigation. In this context, real-world clinical experiences such as those reported by Santamaria et al. (19) may provide valuable information regarding feasibility, safety, and preliminary signals of efficacy while higher-level confirmatory studies are being developed.

Particularly important will be comparative evaluation of the different regenerative strategies currently available, including PRP alone, cell-based therapies, stem cell–derived products, and combined approaches involving cell mobilization. Such studies will help determine whether the biological hypotheses proposed in this field ultimately translate into reproducible clinical benefits and which interventions are most appropriate for specific patient profiles.

In parallel, several ongoing and recently registered clinical trials are expected to provide higher-quality evidence regarding the efficacy and safety of ovarian regenerative interventions, particularly intraovarian PRP. These studies are anticipated to improve patient selection criteria, standardize PRP preparation protocols, define clinically meaningful reproductive endpoints, and clarify the biological mechanisms underlying treatment response. Their results will be essential for determining whether the promising biological rationale supporting ovarian regenerative therapies ultimately translates into reproducible clinical benefit.

### 7.4 Regulatory and safety considerations

The rapid expansion of regenerative therapies in reproductive medicine raises important regulatory and ethical challenges. Although PRP offers advantages related to its autologous nature and generally favorable safety profile, the absence of standardized protocols and definitive clinical evidence warrants continued caution regarding its widespread implementation (14, 35).

For cell-based therapies and progenitor mobilization strategies, regulatory considerations become even more complex. Cellular manipulation, characterization of advanced biological products, and assessment of potential risks require rigorous regulatory frameworks capable of ensuring intervention quality and patient safety (44).

Another particularly important issue is the need to avoid generating unrealistic expectations among patients with poor reproductive prognosis. The concept of ovarian rejuvenation has attracted substantial scientific and public interest; however, current evidence does not support the conclusion that these interventions consistently reverse the fundamental mechanisms of ovarian aging or fully restore lost fertility (7, 13).

In this context, responsible development of the field will require a combination of basic research, rigorous clinical validation, and appropriate regulatory oversight. Only through such an integrated approach will it be possible to determine the true role that PRP, stem cell mobilization, and combined regenerative strategies may ultimately play within the future of reproductive medicine.

Commercial CE-marked devices are currently available for PRP preparation. However, regulatory approval of collection devices should not be interpreted as approval of intraovarian PRP as an evidence-based therapeutic indication, which remains investigational.

## 8 Conclusions

Ovarian rejuvenation represents one of the most dynamic and debated fields in contemporary reproductive medicine. Although platelet-rich plasma (PRP) has emerged as one of the most extensively investigated biological approaches for attempting to restore ovarian function, the available clinical evidence remains heterogeneous and does not yet support definitive conclusions regarding its efficacy across all clinical settings (16, 25, 35, 36).

Advances in the understanding of ovarian aging have promoted a progressive shift in scientific perspective. Rather than considering the follicle as the sole therapeutic target, current evidence suggests that reproductive function depends largely on the integrity of the ovarian niche, a complex microenvironment whose deterioration contributes to the progressive decline of ovarian reserve and follicular competence. From this perspective, regenerative interventions should aim not only to stimulate residual follicles but also to restore the biological conditions required for their survival, development, and function (1–3, 31).

Within this framework, both cell-based therapies and peripheral blood–derived biological products appear to share a mechanism of action predominantly mediated through paracrine modulation of the tissue microenvironment. Their effects on angiogenesis, inflammation, stromal remodeling, and intercellular communication provide a biologically plausible basis for the partial recovery of residual ovarian function reported in selected experimental and clinical studies (8, 9, 18, 29).

Hematopoietic mobilization represents a particularly intriguing approach within this conceptual framework. In addition to increasing the availability of circulating progenitor cells and modulating mechanisms involved in tissue repair and angiogenesis, it may contribute to reshaping the systemic biological environment upon which regenerative interventions act (26, 27, 33, 39). An important unresolved question is whether the various autologous biological preparations used in regenerative medicine truly possess equivalent biological properties, or whether interventions such as cell mobilization can alter their composition and regenerative potential. This possibility challenges the common assumption that PRP represents a biologically uniform intervention and suggests that functional characterization of autologous products may become a key element in the future development of regenerative reproductive medicine (14, 15, 19). Clarifying this issue may help explain part of the variability observed among clinical studies and open new opportunities for the development of more personalized regenerative therapies.

Although rigorous clinical trials, better-defined biomarkers, and standardized procedures will be required to validate these hypotheses, the integration of local and systemic regenerative signals represents one of the most compelling avenues for future investigation. Rather than focusing exclusively on increasing the number of available oocytes, this approach seeks to restore ovarian niche functionality as a prerequisite for preserving or recovering reproductive competence (7, 19). In this regard, the transition from a follicle-centered paradigm toward an ovarian niche–centered paradigm may represent one of the most significant conceptual developments in the recent evolution of regenerative reproductive medicine.

Ultimately, the future of ovarian rejuvenation may depend less on the isolated activation of residual follicles and more on the ability to restore, in an integrated manner, the biological ecosystem that sustains them.

Finally, the future development of ovarian regenerative medicine will require rigorous mechanistic investigation, standardized biological characterization of regenerative products, transparent reporting of clinical protocols, and adequately powered randomized controlled trials to distinguish biologically plausible hypotheses from evidence-based therapeutic strategies. Until such evidence becomes available, ovarian regenerative interventions should be considered investigational.
